# Intermittent Noise Induces Physiological Stress in a Coastal Marine Fish

**DOI:** 10.1371/journal.pone.0139157

**Published:** 2015-09-24

**Authors:** Tye A. Nichols, Todd W. Anderson, Ana Širović

**Affiliations:** 1 Department of Biology and Coastal and Marine Institute, San Diego State University, San Diego, California, United States of America; 2 Scripps Institution of Oceanography, University of California San Diego, La Jolla, California, United States of America; University of Auckland, NEW ZEALAND

## Abstract

Anthropogenic noise in the ocean has increased substantially in recent decades, and motorized vessels produce what is likely the most common form of underwater noise pollution. Noise has the potential to induce physiological stress in marine fishes, which may have negative ecological consequences. In this study, physiological effects of increased noise (playback of boat noise recorded in the field) on a coastal marine fish (the giant kelpfish, *Heterostichus rostratus*) were investigated by measuring the stress responses (cortisol concentration) of fish to increased noise of various temporal dynamics and noise levels. Giant kelpfish exhibited acute stress responses when exposed to intermittent noise, but not to continuous noise or control conditions (playback of recorded natural ambient sound). These results suggest that variability in the acoustic environment may be more important than the period of noise exposure for inducing stress in a marine fish, and provide information regarding noise levels at which physiological responses occur.

## Introduction

The importance of naturally occurring sound has been demonstrated for a variety of marine organisms through its influence on ecological processes. Such processes include orientation toward suitable habitat during settlement of fishes [1,2] and invertebrates [3], the timing of settlement [4] and metamorphosis [5] of invertebrates, and the reproductive behavior of fishes involving sound production [6,7]. The use of sound by many aquatic organisms is not surprising considering the efficiency with which it transmits information underwater. A sound made underwater travels farther, faster, and with less attenuation than an equivalent sound made in air [8], and its propagation is unaffected by the dynamics of water movement [9]. From a sensory perspective, this suggests that sound has the potential to be very useful to aquatic organisms. Conversely, when considering anthropogenic noise as a pollutant of natural habitats, these physical properties could make noise pollution highly disruptive in aquatic environments.

Anthropogenic noise in marine habitats has increased considerably in recent decades due to various activities, including offshore construction, resource acquisition, sonar emissions, and shipping and boating [10–12]. Until recently, research on this issue has primarily explored effects on cetaceans, including interference of acoustic communication, disruption of social structure that is dependent on communication, and mortality due to navigational disruption and strandings (reviewed in [13]). We know relatively little, however, about whether and how noise pollution affects other marine organisms [12].

Collectively, motorized vessels produce what is likely the most common form of noise pollution in the ocean [11,14]. The spectral levels produced by most boat engines are highest in the low to mid frequency range (20–1,500 Hz), with small vessels producing peak levels at higher frequencies than large vessels [15,16]. Nearly all marine fishes that have been evaluated for hearing capabilities are most sensitive to sounds occurring in a low to mid frequency range (20–1000 Hz; [17,18]), indicating considerable spectral overlap between the frequency of peak noise levels produced by boats and the sensitivity of fish hearing. The amount of boat noise in heavily populated regions has risen steadily with increased human activity [12], and reports collected from areas near California suggest that anthropogenic noise may have increased by more than threefold since pre-industrial conditions [19], highlighting a need to better understand whether and how these changes in the acoustic environment may affect fishes.

One of the ways anthropogenic noise can directly affect animal physiology is by inducing stress [20–22]. Measurement of the stress hormone cortisol, a glucocorticoid produced through stimulation of the hypothalamo-pituitary-interrenal axis, is one of the most widely accepted methods in evaluating the stress response of fishes [23,24]. Cortisol is known to mediate metabolic changes triggered by a stressful event [23]. During a stress response, energy stored in lipids can be broken down and made immediately available in the form of blood glucose, which may build up in the circulatory system [25] or be quickly depleted if physical exertion also occurs during a behavioral response to the stressor [26]. Elevated cortisol levels have been correlated with detrimental effects on fishes, including increased susceptibility to infection [22], decreased growth rates [27,28], and reduced predator avoidance ability [29].

Nearly all previous studies investigating stress responses in fishes to anthropogenic noise have used tone generators or machinery associated with aquaculture facilities as the noise source rather than noise a fish may encounter in the wild [21,22,26,30]. One study, however, has examined the cortisol response of fishes to playback of recorded boat noise, finding that intermittent noise caused elevated cortisol levels in three species of freshwater fish while continuous white noise did not [31]. These results suggest that intermittent noise is more stressful for fishes than continuous noise, but because two different forms of noise were used (i.e., boat noise and white noise), there was no direct comparison of stress responses to intermittent versus continuous noise from the same source.

Boat noise is likely to occur in an intermittent temporal pattern [32] as boats move across a given location, whereas other forms of anthropogenic noise are continuous, such as offshore power generators [33]. To our knowledge, direct comparisons of stress responses to intermittent and continuous noise have not been conducted in fishes or other wild animals, and these comparisons may advance our understanding of what specific qualities of anthropogenic noise are responsible for stress induction in the wild. Studies on laboratory animals have indicated that when stressful events occur intermittently, physiological responses may depend on the predictability in timing of these events [34–36].

In addition, there has been no previous research to our knowledge that has directly investigated how stress in fish is affected by differing noise levels, although this has been shown to affect stress responses in laboratory animals [37] and humans [38], with a relatively high level of noise being required to produce measurable changes in stress hormones.

In this study, we investigate the effects of increased noise (playback of boat noise recorded in the field) on the physiological stress response of a common coastal fish, juvenile giant kelpfish (*Heterostichus rostratus*, family Clinidae). We conducted experiments by obtaining recordings of a boat engine in the field and playing them through underwater speakers into laboratory aquaria containing captive fish. The laboratory setting allowed for careful control of the acoustic environment and precise measurements of the hormonal response that would not have been possible through field experiments. A disadvantage of reproducing sounds in the laboratory is that alteration of the sound from the original source is unavoidable, even when every effort is made to allow the two to be as similar as possible. Sounds projected into an aquarium create complex sound fields that differ from what is created in an open-water environment [39,40], and are also known to produce high levels of particle motion. Therefore, the results from any study that involves the reproduction of underwater sound in a laboratory must be cautiously evaluated in relation to what animals experience in the wild. We do not attempt to use the results of this study to make predictions about how a fish would respond in the wild under specific acoustic conditions. Instead, our objective with this work is to use the controlled environment possible in the laboratory to advance our very limited knowledge of how fishes respond physiologically to anthropogenic noise.

We addressed three questions during this research: (1) does increased noise induce physiological stress in juvenile giant kelpfish? (2) If so, does this stress response depend on whether that noise is continuous or intermittent? (3) How does the stress response change with noise level? We hypothesized that giant kelpfish would exhibit stress responses when exposed to increased noise treatments compared to control conditions, and that more acute stress responses would result from treatments involving intermittent noise exposure because this indicates an unstable and potentially hazardous environment to fish.

## Methods

### Ethics Statement

All procedures involving animal subjects in this study were conducted in accordance with regulations of the San Diego State University Institutional Animal Care and Use Committee (Protocol # 12-03-008A). Juvenile giant kelpfish were housed at a density of up to 20 fish in 80 L glass aquaria, which included simulated eelgrass habitat and a constant supply of filtered seawater. Fish were euthanized using tricaine methanesulfonate (MS-222; 500mg/L) at the conclusion of each stress response trial as was necessary for lipid extraction and cortisol measurement. Collections of all fish were conducted with permission of the California Department of Fish and Wildlife.

### Study species

The giant kelpfish is a macrophyte-associated species commonly found in southern California within kelp forests consisting of giant kelp (*Macrocystis pyrifera*) and in eelgrass (*Zostera marina*) beds [41]. They settle into these habitats at approximately 3–4 cm total length in spring and summer months [41]. For this study, juveniles measuring 4–6 cm standard length (SL) were collected by beach seine in San Diego Bay and Mission Bay. These fish were then transported to the San Diego State University Coastal and Marine Institute Laboratory where they were fed live brine shrimp and housed in laboratory aquaria supplied with flow-through seawater constantly pumped from San Diego Bay, allowing temperatures to reflect those found in the field (18–24°C). Water flowed into aquaria below the surface to minimize noise and the housing area was exposed to ample natural light, allowing fish to remain on a natural light-dark cycle. All fish used in this study were evaluated to be in good health and had not been used in any previous experiments.

### Experimental aquaria

**Fig 1:**
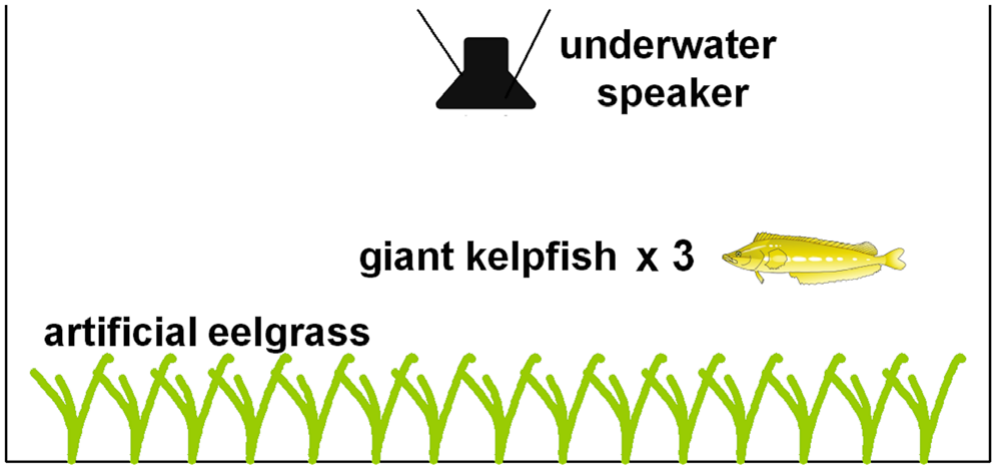
Side view schematic diagram of experimental aquaria setup. Setup for a single replicate used in stress response experiments using an aquarium containing three juvenile giant kelpfish with an underwater speaker suspended from above and artificial eelgrass habitat (fish illustration by L.G. Allen).

Experimental aquaria constructed of glass (width: 30 cm, length: 75 cm, height: 30 cm, 67 L) were used for stress response experiments and arranged in an insulated environmentally controlled room to minimize external noise (Fig 1). Each aquarium contained artificial eelgrass (100 shoots per m^2^) to simulate a low-density eelgrass habitat. Fish were provided with habitat because the absence of shelter may influence stress-related responses [42], which are likely to be relevant for fishes that are strongly associated with habitat structure. Water temperature in experimental aquaria was recorded at the start of each acclimation period and at the conclusion of each trial to ensure that it remained within ~ 1°C of housing conditions. Timed lighting was provided in the room containing experimental aquaria, allowing fish to remain on a natural light-dark cycle after being transferred from housing tanks. Each aquarium was equipped with an underwater speaker (UW-30, flat frequency response: 0.1–10,000 Hz, Electro-voice, Lincoln, NE, USA) powered by an amplifier (40W Portable Amplifier; frequency response: ~ 40–20,000 Hz, Radio Shack, Fort Worth, TX, USA). Speakers were suspended from the top of aquaria and submerged below the water surface to exclude vibrations caused by speaker contact with aquarium surfaces. All sound files used were band-pass filtered between 100–2000 Hz in order to play within speaker specifications and to minimize resonant frequencies within aquaria [39].

### Recordings used for sound treatments

**Fig 2:**
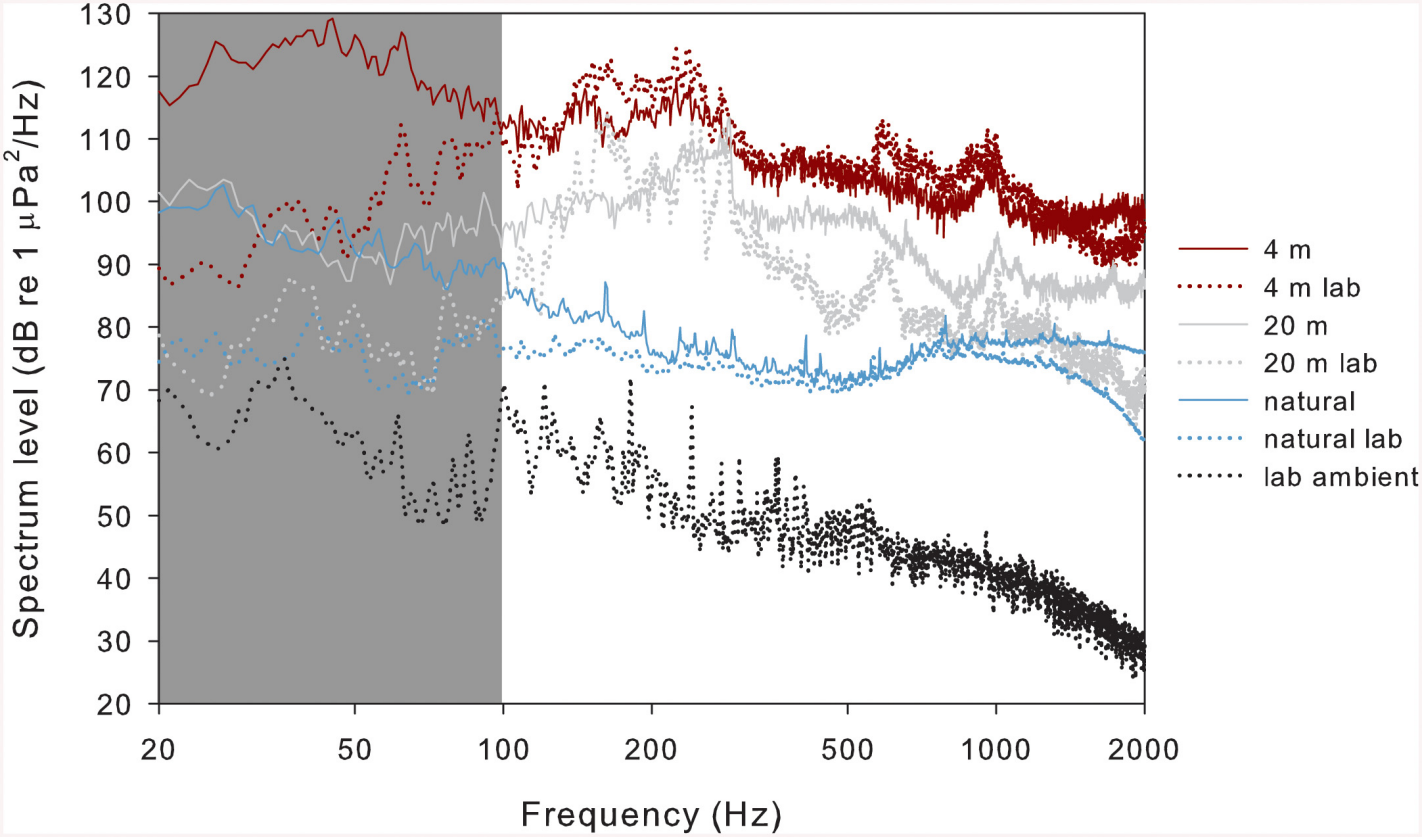
Frequency spectra for example field and laboratory recordings. Sound pressure levels for field recordings and playback of recordings into aquaria for laboratory treatments (Hanning window, FFT length: 48,000, 50% overlap). Solid lines show the spectra for field recordings: solid red, 4 m from accelerating boat; solid grey, 20 m from accelerating boat; solid blue, natural sounds of an eelgrass bed. Dotted lines show the spectra for recordings of band-pass filtered (100–2,000 Hz) playback of field recordings into laboratory aquaria: dotted red, 4 m from accelerating boat; dotted grey, 20 m from accelerating boat; dotted blue, natural sound of an eelgrass bed. The spectrum for ambient sound of aquaria is shown by the dotted black line. The shaded region denotes the low frequencies (0–100 Hz) that were minimized for laboratory playback. All spectra represent an average of multiple recordings for each type.

Boat noise recordings were made in the vicinity of a boat with an outboard boat engine (175 hp, Yamaha Motor Co., Ltd.) in San Diego Bay. Multiple recordings of noise were taken at each of several distances from the hydrophone to the boat engine (4, 6, 8, 10, 15 and 20 m), which represented a range of noise levels from very loud at 4 m to only moderately audible at 20 m (Fig 2; S1 Fig). Water column depth was 6–10 m and the hydrophone was positioned at a depth of 1.5 m. For each recording, the boat started with the engine at the measured distance from the hydrophone and then accelerated away from the hydrophone to allow precise measurements of the boat engine starting distance. At least three separate recordings were obtained for each distance for use in laboratory experiments to account for any variations in acceleration rate of the boat and ambient noise during recordings. For control treatments, natural ambient sound was recorded from eelgrass beds where giant kelpfish were collected to represent the acoustic environment that these fish would experience in the absence of anthropogenic noise (Fig 2). These recordings were made in a shallow eelgrass bed in Mission Bay at Mariner’s Basin (32°45ʹ55.21ʺN, 117°14ʹ47.60ʺW). Recordings were taken from several locations of the eelgrass bed at depths ranging from 0.5–1.5 m. The area was chosen for low human activity, and no boating or other activities were observed in the vicinity of the eelgrass bed during recordings. These recordings were dominated by the sounds of snapping shrimp (*Alpheus* spp.) along with various other sounds, likely produced by invertebrates.

All recordings were collected using an HTI-96-MIN hydrophone with a built-in preamplifier (sensitivity: –164 dB re 1 μPa; frequency range 2–30,000 Hz, High Tech Inc., Long Beach, MI, USA) and a 16-bit digital Edirol R-09HR recorder (Roland, Los Angeles, CA, USA) sampling at 48 kHz. The recorder was calibrated using pure sine wave signals of known frequency and intensity across the frequency range of interest (20–2,000 Hz). This frequency range extends across the suspected hearing sensitivity range of the study species based on data collected from other fishes [18] and covered the frequencies analyzed for subsequent calculations. Prior to conducting trials, all sound treatment files were played using a portable player (8K804QK; frequency response: ~ 40–20,000 Hz, Apple, Cupertino, CA, USA) through speakers into experimental aquaria and re-recorded using the same hydrophone and recorder to compare peak-to-peak intensities and spectral levels to the original recordings (Fig 2; S2 and S3 Figs). Adjustments to spectral characteristics were made using a parametric equalizer in Sonar v. 6 (Cakewalk, Boston, MA, USA), and adjustments to the sound level of playback were made on the amplifier so that sounds occurring in aquaria would be as similar as possible to field recordings. Spectral levels of recordings were calculated using a fast-Fourier transform (FFT) and a Hanning window with an FFT length of 48,000 samples and 50% overlap using Triton v. 1.8 ([43]; Fig 2; S1–S3 Figs). Root-mean-square (rms) sound pressure levels (SPLs) of recordings of playback into experimental aquaria were also calculated over the calibrated frequency range from averaging pressure values of multiple recordings for each type.

### Stress responses to continuous vs. intermittent noise

To better understand what specific qualities of noise induce stress, an experiment was conducted to compare stress responses in juvenile giant kelpfish (4–6 cm SL) to continuous and intermittent noise. Prior to noise exposure trials, fish were placed in experimental aquaria with underwater speakers using the design described above. Three fish were placed in each aquarium, giving a density for experimental conditions that is within the range of densities that were observed in the field during collections. The size of fish used for each trial was assessed to achieve similarity in size among treatments. Fish were placed into aquaria and acclimated for 20 h during which natural sounds played through the speaker. At the end of the acclimation period fish were exposed to one of the sound treatments. The duration of all trials was 60 min, which was chosen based on the peak in cortisol response observed from a pilot experiment (S1 and S2 Data).

Each increased noise treatment was prepared by using multiple boat noise recordings taken from a distance of 6 m from the hydrophone. The recordings were arranged randomly in a 10–min file using Sonar, which was played through the speaker and looped as necessary for the duration of a trial. Each noise event lasted 5–12 sec with time between noise events varying from 1–120 sec. Recorded natural sound from the eelgrass bed also played continuously throughout noise treatments. For two of these treatments (‘random intermittent noise’ and ‘regular intermittent noise’), noise events occurred intermittently and comprised ~ 40% of the files while recorded natural sounds played continuously throughout, including between noise events. The proportion of increased noise was chosen to approximate a moderate to high traffic environment based on recordings made in an eelgrass bed near Shelter Island (32°42’50.13” N, 117°13’31.29” W) in San Diego Bay in which anthropogenic noise was observed > 50% of the time. In the ‘random intermittent noise’ treatment noise events occurred randomly in time, and in the ‘regular intermittent noise’ treatment noise events occurred in a regular repeating pattern (two noise events occurred in succession lasting 10 sec followed by a 15 sec break). These treatments were used to determine whether predictability in the temporal pattern of noise events would affect stress responses while the total amount of noise exposure remained constant. A third noise treatment (‘continuous noise’) consisted of recorded boat noise without breaks, so that increased noise was present during 100% of the trial along with recorded natural sound. Recorded natural sound was played alone as a control to represent an acoustic environment in the absence of anthropogenic noise. The natural sound treatment was prepared by randomly arranging clips from the collection of recordings from the eelgrass bed to construct a 10-min sound file. The file created for each treatment was used for all trials conducted for that treatment. This was done in order to minimize potentially confounding variables as our intent was to determine whether physiological responses of giant kelpfish would depend on the temporal pattern of noise they were subjected to, not whether they would respond to playback of recorded boat noise in general. One environmentally controlled room was used for conducting all trials with one treatment type randomly selected for each day. Treatments were always conducted separately and fish were never used in multiple trials. Six replicates per treatment were conducted using three fish per replicate.

### Stress responses to different noise levels

To determine whether juvenile giant kelpfish would respond to increased noise over a range of sound levels, fish were exposed to playback of noise that was recorded at multiple distances from the boat engine. Recordings obtained in the field with the engine starting at 4, 6, 8, 10, 15, and 20 m away from the hydrophone were used to create files for playback in the laboratory as increased noise treatments. Each of the six treatments consisted of multiple recordings arranged in a random intermittent pattern of events that was similar to what was used in the previous experiment, and recorded natural sounds were included throughout all files. Two replicates for each noise level were conducted using three fish per replicate on separate days in a randomized order. All other procedures were unchanged from the previous experiment.

### Cortisol extraction and measurement

The protocol for cortisol extraction and measurement was the same for all trials conducted. All sound exposure trials began between 0600 and 0700 to account for possible diel variability in hormone levels [44,45], using three juvenile giant kelpfish per trial. At the conclusion of each trial, fish were captured and euthanized using MS-222. The time elapsed between capture and euthanasia of fish was less than 30 sec to avoid potentially confounding stress responses. Fish were then placed in sealed plastic bags and immediately frozen using a solution of isopropyl alcohol and dry ice (-70°C). All individuals were then stored in a freezer (-20°C) until further processing.

The small size of juvenile giant kelpfish required that cortisol concentrations be measured using whole-body extractions [46] before performing an enzyme-linked immunosorbent assay (ELISA). Length (mm SL) and mass measurements were taken for all frozen individuals before they were ground to a fine pulp using a mortar and pestle. Pulp was then suspended in 5 ml DI water, transferred to a scintillation vial, and stored in a freezer. Lipids were extracted from the contents of each vial using a diethyl ether extraction method [47]. Lipid extract was used to conduct the ELISA according to kit instructions (ELISA Cortisol Kit 900–071; Enzo Life Sciences, Farmingdale, NY, USA). Regression calculations for comparing standards of known cortisol concentration to individual samples were performed automatically using a calibrated microplate reader and accompanying software (SoftMax Pro version 4.0, Sunnyvale, CA, USA). These concentrations were then adjusted according to the mass of each individual. Cortisol concentrations of the three fish used for each trial were averaged to represent a single replicate to account for individual variability in stress response within trials.

### Statistical analysis

Statistical analyses of all results in this study were performed using SYSTAT v. 12 (SYSTAT Inc., Chicago, IL, USA) unless otherwise specified. All data were found to satisfy the assumptions of parametric analyses (S3 and S4 Data). To analyze stress responses to continuous vs. intermittent noise, a one-way ANOVA and Tukey’s HSD multiple comparison test were used to determine whether there was a difference in cortisol concentration among treatments and to determine which treatments differed from each other. For stress responses to different noise levels, a regression (SigmaPlot v. 11, SYSTAT Inc., Chicago, IL, USA) was used to analyze the relationship between the noise level of increased noise playback in aquaria and cortisol concentration in giant kelpfish.

## Results

### Stress responses to continuous vs. intermittent noise

**Fig 3:**
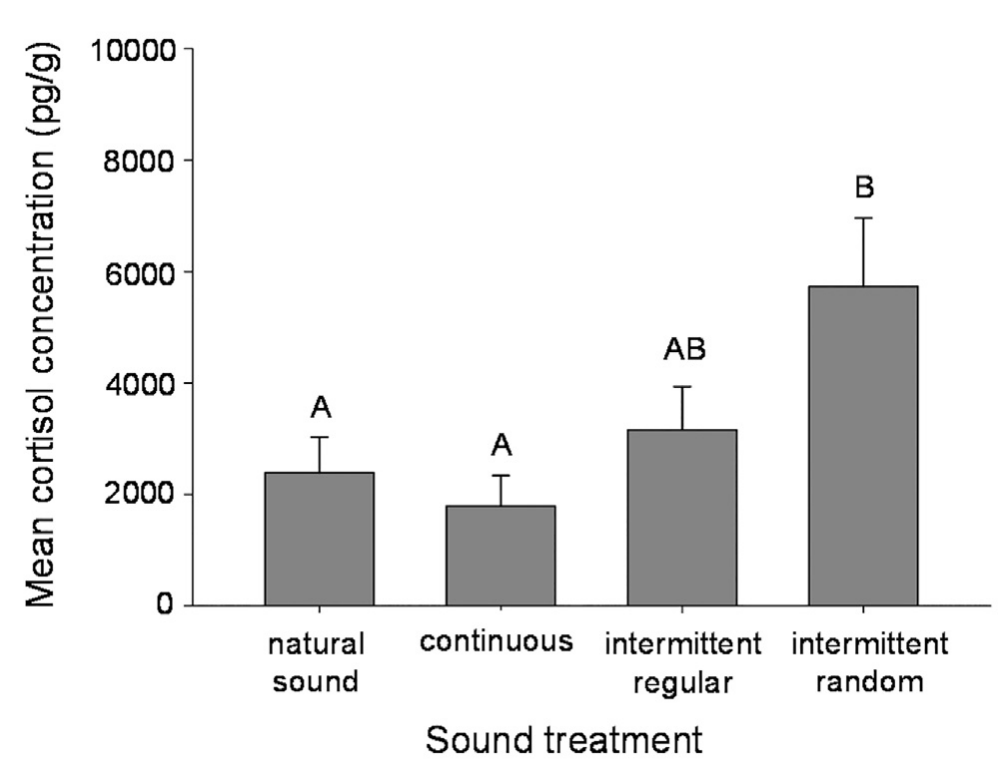
Cortisol response to different temporal patterns of playback of recorded boat noise and natural sound. Mean (+SE) cortisol response of juvenile giant kelpfish using playback of recorded natural sound as a control and different temporal patterns of increased noise. Different letters denote statistically significant differences among treatments based on Tukey’s HSD test. Three fish were used per replicate (n = 6).

Juvenile giant kelpfish exposed to random intermittent noise exhibited significantly higher cortisol concentrations than those that were exposed to continuous noise and control treatments (ANOVA, *F*_3,20_ = 4.27, p = 0.018, Fig 3; S3 Data). Cortisol response to random intermittent noise, however, was not significantly different from regular intermittent noise, which in turn, was not significantly different from any of the other three treatments (Fig 3; S3 Data).

### Stress responses to different noise levels

**Fig 4:**
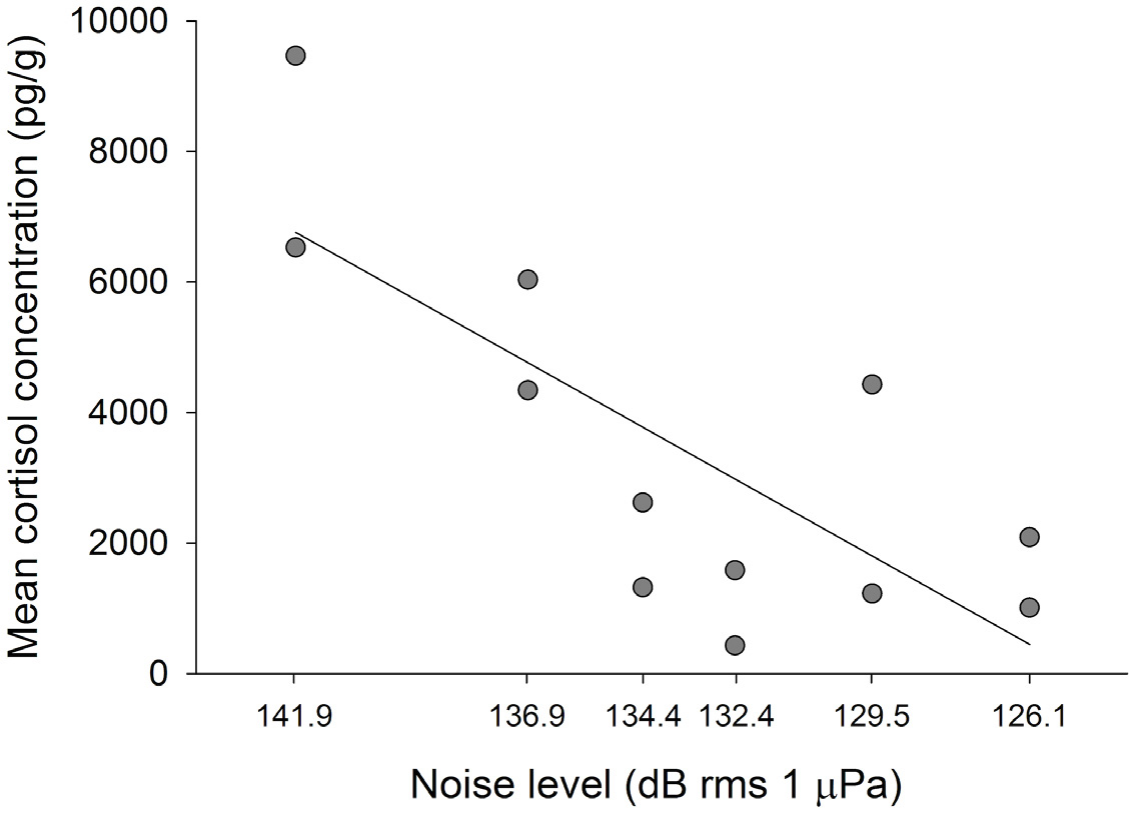
Cortisol response to different noise levels of playback of recorded boat noise. Linear relationship between the noise level of playback of recorded boat noise and the cortisol response of juvenile giant kelpfish. The six treatments were created through playback of multiple boat noise recordings made in the field at each of six distances (4, 6, 8, 10, 15 and 20 m) from the boat with noise level decreasing as distance increased. Each symbol represents the mean cortisol concentration of the three fish used per replicate (n = 2 for each noise level).

Cortisol response of giant kelpfish decreased linearly with the noise level of increased noise treatments (r^2^ = 0.58, *F*_1,10_ = 14.04, p = 0.004, Fig 4; S4 Data). Analyzed and adjusted playback of sound treatments into experimental aquaria produced spectra that are similar to those obtained from field recordings (Fig 2; S1–S3 Figs), although it is not possible for sounds reproduced in aquaria to perfectly match those obtained in the field. Some differences that can be observed include a larger frequency range of peak SPL for playback of boat noise recorded at a distance of 20 m and a less dynamic spectrum for playback of recorded natural sounds when these are compared to the original field recordings (Fig 2). For field recordings of boat noise taken from 4 and 6 m the peak SPL was close to 50 Hz, but for all greater distances the peak occurred between 200–400 Hz (S1 Fig). Laboratory playback of these recordings all showed peak SPLs between 200–400 Hz due to the high-pass filter that was necessary for recordings to play within the specifications of the speaker (S2 Fig).

## Discussion

This study demonstrates that the occurrence and magnitude of the stress response of giant kelpfish to increased noise depends on the temporal pattern and level of noise events, with intermittent noise of high SPL inducing the greatest response. Results of this work provide direct evidence to support the hypothesis that intermittent noise can be more stressful to a fish than the same noise when it is continuous, which has been suggested by previous studies [21,31]. This research extends a growing body of work in examining the various ways in which anthropogenic noise can impact fishes, which include inducing physiological stress [21, 31], altering heart rate [48], and reducing foraging success [49].

The mechanisms involved in the physiological stress response of fishes are part of a complex and adaptive system, which has evolved to maintain homeostasis when a fish is confronted with various environmental changes (reviewed in [50]). Giant kelpfish exposed to intermittent noise exhibited an acute stress response while those exposed to continuous noise did not, even though fish subjected to continuous noise experienced more than twice the duration of noise exposure during each trial. Previous research has suggested that fish may habituate more quickly to continuous noise because it produces more constant acoustic conditions, whereas intermittent noise that produces fluctuations in the stimulus could induce greater stress responses [21,31] and prolong altered behavior which is likely related to the physiological response [51]. This is also supported by studies that have reported limited effects on fishes exposed to the continuous noise of aquaculture facilities [30] and offshore wind farms [33]. These findings along with the results of the present study suggest that fluctuation in the acoustic environment may mediate the physiological response for a variety of fishes, potentially because greater instability in a soundscape signals a hazardous environment.

Research on laboratory rats has shown that when stressful events occur intermittently, responses are more acute when events occur in an unpredictable or random pattern versus a predictable or regular pattern [34–36]. In our experiment, manipulating the predictability in timing of noise events did not produce clear results, as the mean cortisol level of fish exposed to a regular pattern of noise was not statistically different from that of a random pattern or any other treatment. However, that fish exposed to a random pattern of noise showed significantly higher cortisol levels than for continuous noise or natural sounds while fish exposed to a regular pattern were not different from other treatments suggests that predictability in the timing of noise events may be important, and that lower predictability may induce higher stress.

The physiological effects of noise on an organism are likely to depend partly on the magnitude of the noise, although this relationship has rarely been investigated. The cortisol concentration of giant kelpfish was elevated only in response to playback of noise recorded at the smallest distances from the boat engine (4 and 6 m), which produced the highest SPLs inside aquaria (141.9 and 136.9 dB rms re 1 μPa, respectively). This is comparable to the results found in laboratory rats [37], which are the only other animal to our knowledge that have been tested similarly. The giant kelpfish did not respond physiologically to playback of boat recordings at lower noise levels. This was indicated by cortisol responses that were comparable to those obtained from fish in control treatments in our other experiments, but this does not rule out potential effects of lower noise levels on fish behaviors such as those involved in social interactions [52] or foraging activity [49] that were not measured here.

It is important to note that playback of boat noise recordings into aquaria cannot reliably simulate what a fish experiences in the field when exposed to boat noise. All fish are likely to perceive the particle motion component of sound [53], which is expected to occur at higher levels in an acoustic environment created by playback of sounds into an aquarium compared to open water. Differences in frequency spectra were also observed between recordings taken in the field and playback of those recordings used for experiments (Fig 2; S1–S3 Figs). Therefore, results given in this study cannot be used to directly determine how a fish would respond to boat noise in the wild. These results may, however, be used to advance a more fundamental understanding of how fish respond physiologically to anthropogenic noise—a subject that we have very limited knowledge of despite that wild fishes may be confronted with noise pollution as much as any other anthropogenic stressor [12]. Advancing this understanding further will require both laboratory and field studies to balance the level of control and manipulation possible in the laboratory with the environmental conditions that can only be attained in the field.

Motorized vessels are likely the most ubiquitous source of anthropogenic noise in all aquatic habitats, altering the acoustic environment particularly in coastal regions where human activity is concentrated. Boat noise is most likely to occur in an intermittent and random temporal pattern as different vessels move across a given location [32]. Results of this study indicate that exposure to intermittent noise induces stress in a common coastal fish, but exposure to the same noise when it is continuous does not induce stress. Stress may be particularly detrimental for juvenile fishes as it can negatively affect growth [27,28] and subsequently increase size-dependent predation risk [54]. Stress can also have more immediate consequences for survival by increasing predation risk shortly after a stressful event [29]. As a multitude of noise-generating human activities (e.g., boating, offshore construction, seismic exploration, sonar activity) continue to expand into marine environments, advancing our knowledge of whether different types of noise affect the physiology and behavior of marine fishes and other organisms will be an important part of impact assessment.

## Supporting Information

S1 Fig Spectra of field recordings of boat noise over a range of distances from the boat. (TIF)

S2 Fig Spectra of playback of field recordings of boat noise into laboratory aquaria. (TIF)

S3 Fig Spectra of field recordings of boat noise and playback into laboratory aquaria. (TIF)

S1 Data Cortisol responses to increased noise or a control for different time periods. (XLS)

S2 Data Cortisol responses to increased noise or a control for 60 or 90 min. (XLS)

S3 Data Cortisol responses to increased noise of different temporal patterns or a control. (XLS)

S4 Data Cortisol responses to increased noise over a range of noise levels. (XLS)
