# Supplementary figures and images for: Intermittent Noise Induces Physiological Stress in a Coastal Marine Fish
Source: PLoS One. 2015 Sep 24;10(9):e0139157. doi: 10.1371/journal.pone.0139157 (PMC4581866; doi:10.1371/journal.pone.0139157)

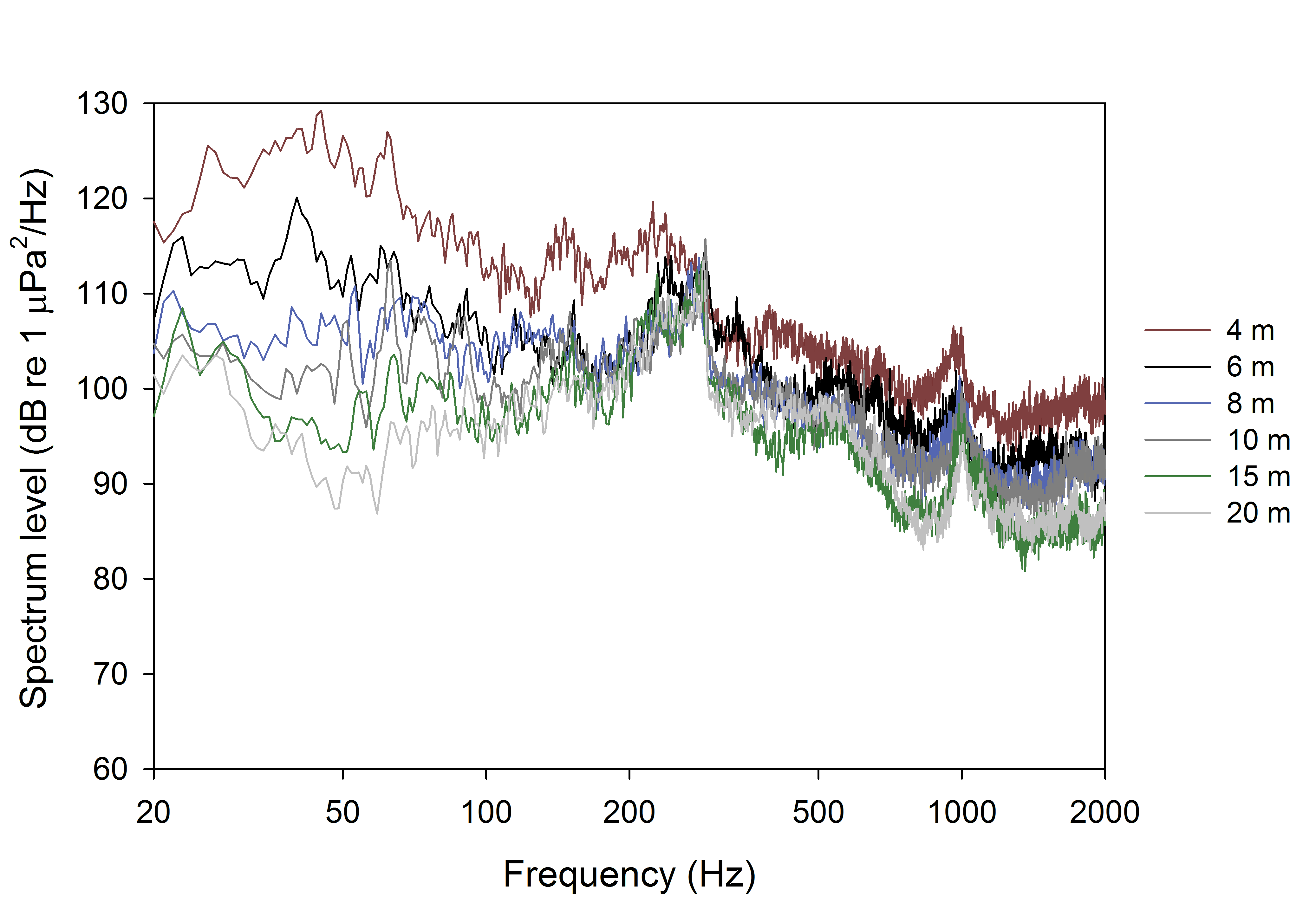

Supplement: S1 Fig — (TIF) [file pone.0139157.s001.tif]

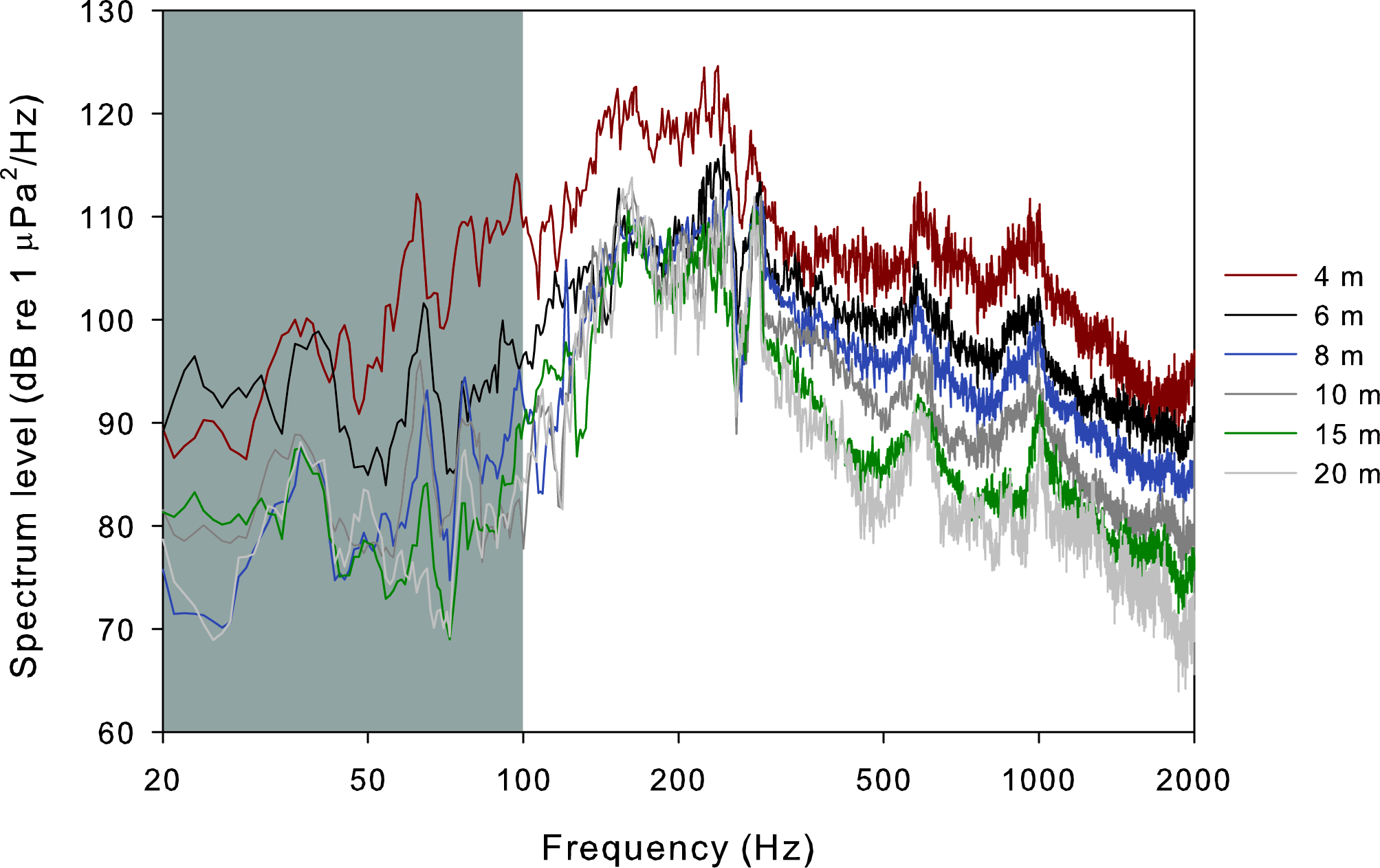

Supplement: S2 Fig — (TIF) [file pone.0139157.s002.tif]

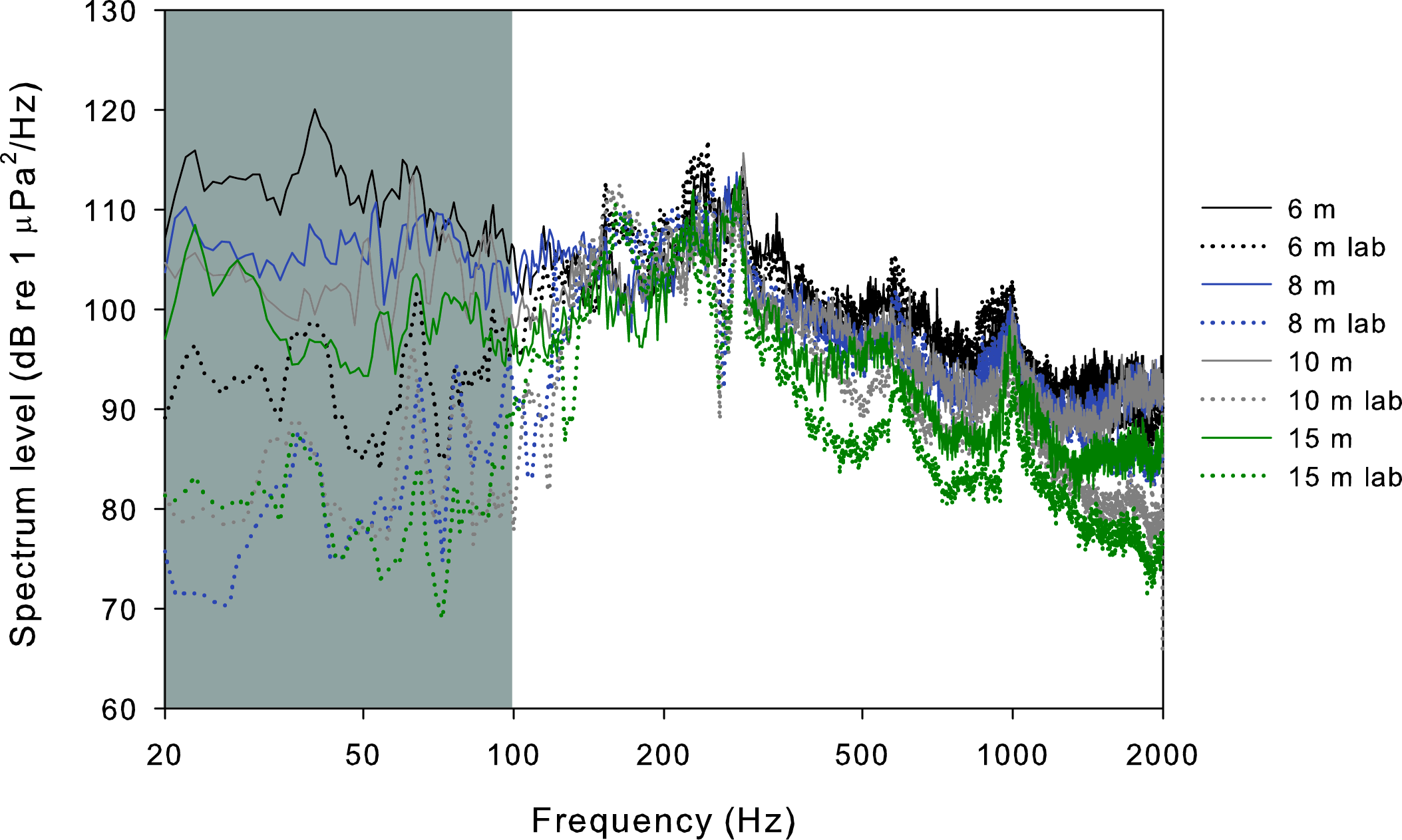

Supplement: S3 Fig — (TIF) [file pone.0139157.s003.tif]
